# Unveiling the Molecular Mechanisms Regulating Muscle Elasticity in the Large Yellow Croaker: Insights from Transcriptomics and Metabolomics

**DOI:** 10.3390/ijms252010924

**Published:** 2024-10-11

**Authors:** Mengyang Liu, Guangde Qiao, Yabing Wang, Shengyu Liu, Xiaoshan Wang, Yanfeng Yue, Shiming Peng

**Affiliations:** East China Sea Fisheries Research Institute, Chinese Academy of Fishery Sciences, Shanghai 200090, China

**Keywords:** *Larimichthys crocea*, muscle quality, association analysis, amino acid metabolism

## Abstract

The large yellow croaker (*Larimichthys crocea*) is an important economic fish in China. However, intensive farming practices, such as high stocking densities, suboptimal water quality, and imbalanced nutrition, have led to a decline in muscle quality. Muscle elasticity is a key texture property influencing muscle quality. Herein, transcriptomic and metabolomic analyses were performed on four groups: male high muscle elasticity (MEHM), female high muscle elasticity (MEHF), male low muscle elasticity (MELM), and female low muscle elasticity (MELF), to explore the molecular regulation underlying muscle elasticity in the large yellow croaker. Transcriptomics identified 2594 differentially expressed genes (DEGs) across the four groups, while metabolomics revealed 969 differentially expressed metabolites (DEMs). Association analysis indicated that the valine, leucine, and isoleucine biosynthesis pathways were significantly enriched between the MELF and MEHF groups; 2-Oxoisovalerate and L-Valine were DEMs; and the gene encoding L-threonine ammonia-lyase was a DEG. In the MELM and MEHM groups, pathways such as arginine biosynthesis; arginine and proline metabolism; and valine, leucine, and isoleucine degradation were significantly enriched. 4-guanidinobutanoate, L-aspartate, N-acetylornithine, and L-leucine were among the DEMs, while the DEGs included *glul*, *gls*, *srm*, *hmgcs*, and *aacs*. These findings provide insights into the molecular mechanisms controlling muscle elasticity, representing a theoretical foundation to breed high-quality large yellow croakers.

## 1. Introduction

The flesh of the large yellow croaker (*Larimichthys crocea*) is tender and flavorful, primarily due to its rich content of proteins, essential amino acids (such as arginine, valine, and leucine), and unsaturated fatty acids (including Omega-3 and Omega-6 fatty acids) [1,2]. Therefore, this fish has significant economic value. It is the most productively farmed marine fish in China, and is one of the eight advantageous aquaculture export products. Before the 1980s, domestic scientists focused their research on species issues, resource changes, and resource conservation of *L. crocea* [3,4]. With the drastic decline of wild *L. crocea*, the research focus has gradually shifted to the artificial breeding and culture of *L. crocea* [5,6,7]. After more than 20 years of such research, *L. crocea* resources have been effectively protected. However, inbreeding between generations over a long period of time has led to problems, such as quality deterioration and decreased disease resistance, which have seriously constrained the further development of the *L. crocea* aquaculture industry. Therefore, it is vital to carry out germplasm improvement and genetic selection of *L. crocea*.

Muscles are the most important edible parts of a fish, and muscle quality directly determines its economic value. The concept of muscle quality is highly complex, encompassing various factors, such as genetic background, farming environment, aquaculture practices, and processing methods. Current research on fish muscle quality has primarily focused on textural properties, nutritional composition, flavor, and muscle fiber structure. For example, freeze–thaw cycles reduce the firmness, elasticity, and chewiness, while increasing the stickiness of halibut (*Thunnus obesus*) flesh [8]. Supplementing feeds with essential amino acids, such as threonine, methionine, and glutamic acid, can significantly enhance the content of crude protein, crude fat, and certain amino acids in fish muscle [9,10,11]. Amino acids, such as glutamic acid, aspartic acid, and lysine, play an important role in fish flavor [12]. The type and content of fatty acids also significantly influence flavor, with wild fish having higher levels of eicosapentaenoic acid (EPA), docosahexaenoic acid (DHA), and n-3 polyunsaturated fatty acids compared with those in farmed fish, leading to a superior flavor [13,14]. Furthermore, the texture of fish is closely related to muscle fiber characteristics, with smaller and denser muscle fibers contributing to more tender meat [15,16].

Textural properties are critical indicators of muscle quality, providing objective measures to assess the freshness and sensory attributes of fish. These properties include hardness, elasticity, gumminess, chewiness, and juiciness. For example, substituting fermented soybean meal for fish meal influenced the muscle hardness and chewiness of Japanese seaperch (*Lateolabrax japonicus*) [17]. Additionally, incorporating appropriate levels of threonine into the diet has been found to enhance the textural properties of carp (*Cyprinus carpiovar* Jian) flesh [18].

In recent years, transcriptomics and metabolomics have become important tools in aquaculture research, particularly to study fish muscle quality. For example, transcriptomic analysis of grass carp (*Ctenopharyngodon idella*) and crisp grass carp (*Ctenopharyngodon idellus*) identified 231 differentially expressed genes (DEGs), including 30 DEGs that were strongly associated with muscle fiber differentiation, extracellular matrix deposition, and muscle hardness [19]. In another study, wild carp muscle was shown to have significantly higher hardness, chewiness, and shear strength than pond carp muscle. Transcriptomic analysis between these two populations revealed that upregulated DEGs in wild carp were primarily enriched in pathways related to the cell cycle, immune regulation, and inositol phosphate metabolism, while downregulated DEGs were associated with RNA transport, carbohydrate metabolism, and protein and lipid metabolism [20]. Moreover, transcriptomic and metabolomic analyses have shown that the addition of hydroxyproline to the diet can alter muscle metabolism and promote collagen synthesis in large yellow croaker, thereby improving muscle quality [21]. In this study, we utilized transcriptomic and metabolomic analyses to determine the molecular regulatory mechanisms of muscle elasticity in the large yellow croaker, aiming to provide a theoretical foundation for breeding high-quality large yellow croakers.

## 2. Results

### 2.1. Transcriptome Analysis

#### 2.1.1. Transcriptome Data Processing and Quality Control

The transcriptome sequencing results for large yellow croaker muscle tissue showed that a total of 75.40 Gb of raw data was obtained, and after filtration, 73.60 Gb of clean data was obtained. Base quality assessment showed that the average Q20% was 96.84% and the average Q30% was 94.37%, indicating high sequencing quality, making it suitable for further analysis (Table 1).

To ensure high-quality sequencing data, raw data were processed, resulting in an average sample alignment to the reference genome of 96.22%, with a minimum alignment of 95.67% (Table 2). Gene coverage analysis demonstrated uniform sequence distribution across genes, with no significant bias, meeting the requirements for further analysis.

#### 2.1.2. Analysis of DEGs

**Figure 1:**
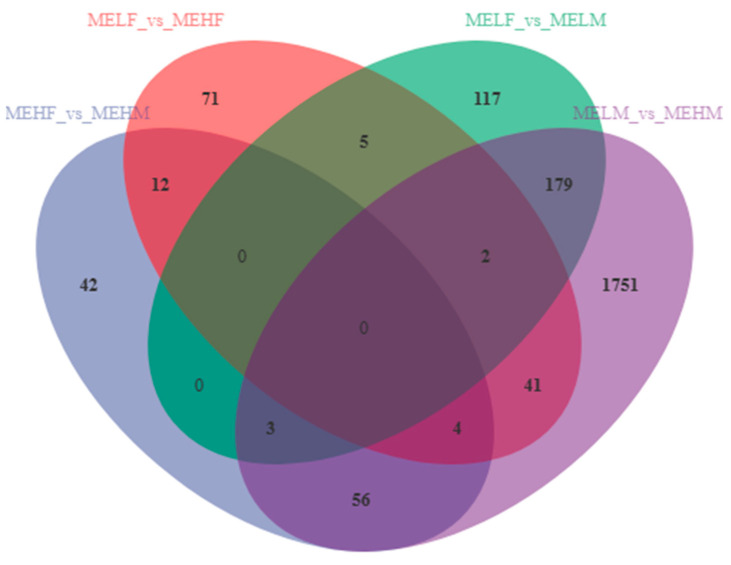
Venn diagram illustrating counts of DEGs in muscle tissue of large yellow croaker (*L. crocea*) with different muscle elasticity. DEG, differentially expressed gene; MEHM, male high muscle elasticity; MEHF, female high muscle elasticity; MELM, male low muscle elasticity; MELF, female low muscle elasticity.

**Figure 2:**
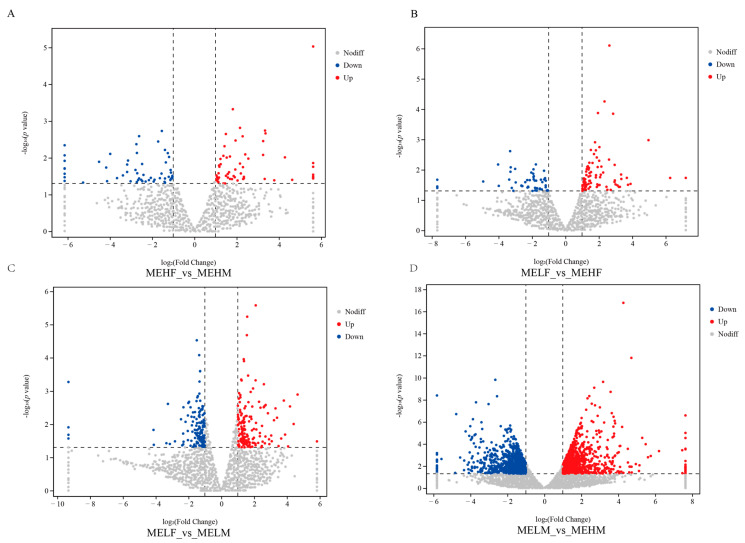
Volcano plot illustrating upregulated and downregulated DEGs in muscle tissue of large yellow croaker (*L. crocea*) with different muscle elasticity. NoDiff, no difference; up, upregulated; down, downregulated. (**A**) Comparison between the female high muscle elasticity group and the male high muscle elasticity group. (**B**) Comparison between the female low muscle elasticity group and the female high muscle elasticity group. (**C**) Comparison between the female low muscle elasticity group and the male low muscle elasticity group. (**D**) Comparison between the male low muscle elasticity group and the male high muscle elasticity group.

Transcriptomic analysis identified 2594 DEGs in the large yellow croaker (Figure 1). In the MEHF_vs._MEHM comparison, 117 DEGs were identified, with 60 upregulated and 57 downregulated. The MELF_vs._MEHF comparison revealed 135 DEGs, with 82 upregulated and 53 downregulated (Figure 1 and Figure 2). The MELF_vs._MELM comparison found 306 DEGs, with 164 upregulated and 142 downregulated. The MELM_vs._MEHM comparison identified 2036 DEGs, with 1152 upregulated and 884 downregulated (Figure 1 and Figure 2).

### 2.2. Validation of the qRT-PCR Data

**Figure 3:**
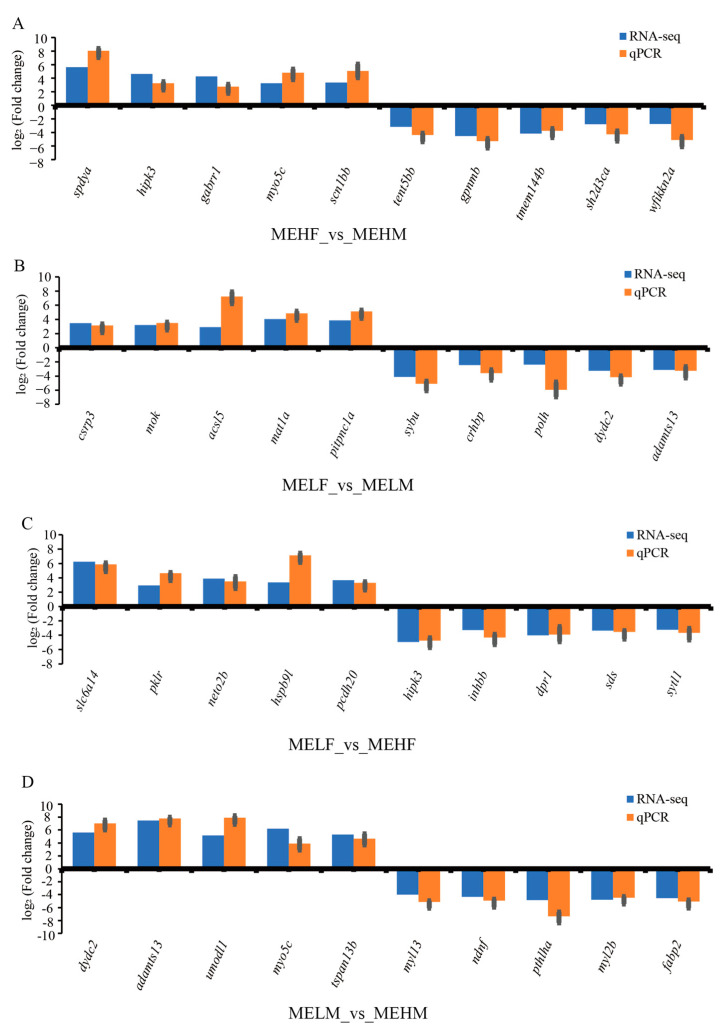
Validation of the relative log_2_ (fold change) between RNA-seq and qPCR when comparing the groups with different muscle elasticity, using *β-actin* expression for normalization. (**A**) Comparison between the female high muscle elasticity group and the male high muscle elasticity group. (**B**) Comparison between the female low muscle elasticity group and the male low muscle elasticity group. (**C**) Comparison between the female low muscle elasticity group and the female high muscle elasticity group. (**D**) Comparison between the male low muscle elasticity group and the male high muscle elasticity group.

As can be seen from the validation results of the transcriptome qRT-PCR data and the validation results of the four comparison groups, RNA-seq coincided with the up- and downregulation trend in qPCR, and the FC values were all relatively close, thus indicating that the transcriptomic data in this study are reliable (Figure 3).

### 2.3. Metabolome Analysis

#### 2.3.1. Multivariate Statistical Analysis

**Figure 4:**
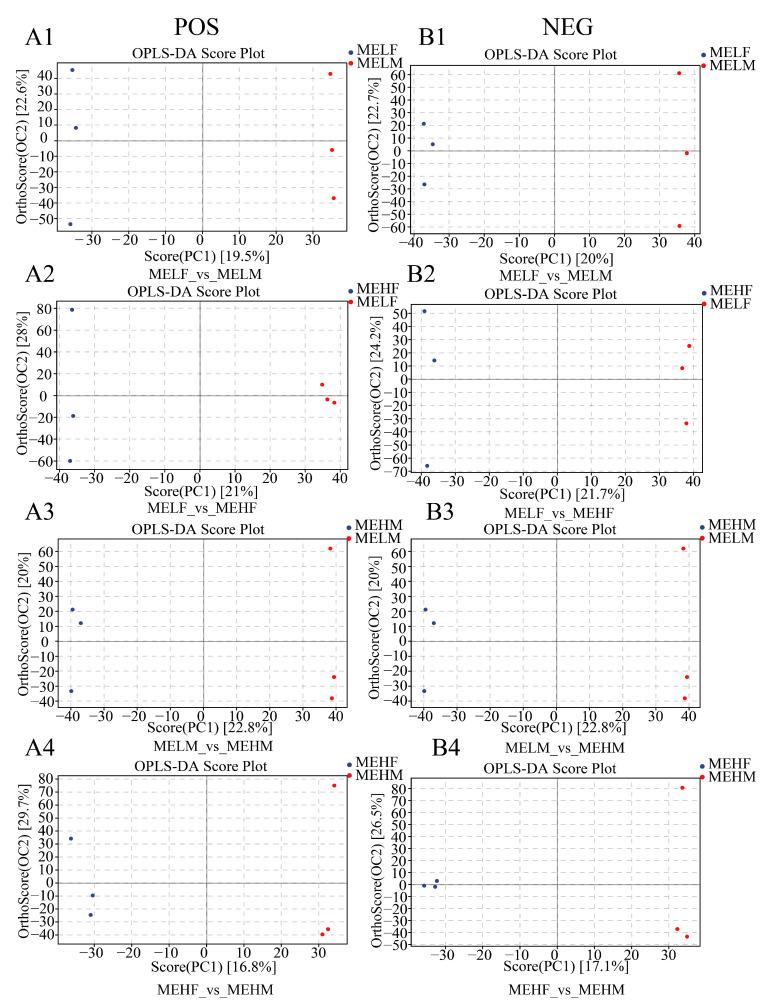
OPLS-DA illustrating the intergroup differences in metabolites among the four comparison groups of large yellow croaker (*L. crocea*) with different muscle elasticity. (**A1**–**A4**) Positive ion mode. (**B1**–**B4**) Negative ion mode. PC1 represents Principal Component 1 and OC2 represents Principal Component 2, each dot represents one sample, and dots of different colors indicate different subgroups. OPLS-DA, orthogonal partial least squares discriminant analysis.

Further analysis using OPLS-DA demonstrated clear separation between the groups in the metabolic spectrum with no overfitting, indicating good model robustness (Figure 4).

#### 2.3.2. Analysis of DEMs

**Figure 5:**
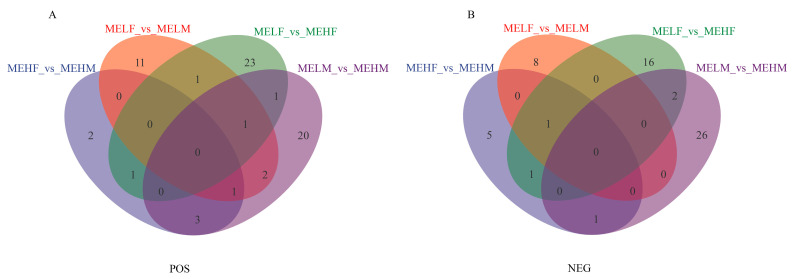
Venn diagram illustrating counts of DEMs in muscle tissue of large yellow croaker (*L. crocea*) with different muscle elasticity. (**A**) Positive ion mode. (**B**) Negative ion mode. DEM, differentially expressed metabolite.

A total of 969 metabolites were identified across both positive and negative ion modes, including 479 in the positive ion mode and 490 in the negative ion mode (Table 3). In the MELF_vs._MELM, MELF_vs._MEHF, MELM_vs._MEHM, and MEHF_vs._MEHM comparisons, 9, 9, 9, and 2 upregulated metabolites were identified, respectively, and 7, 18, 17, and 5 downregulated metabolites were identified, respectively, in the positive ion mode. In the negative ion mode, 3, 8, 14, and 4 upregulated metabolites were identified, respectively, and 6, 12, 15, and 4 downregulated metabolites were identified, respectively (Table 3 and Figure 5).

### 2.4. Findings from Metabolome and Transcriptome Association Analysis

#### 2.4.1. Transcriptional and Metabolomic Co-Enriched Pathways

**Figure 6:**
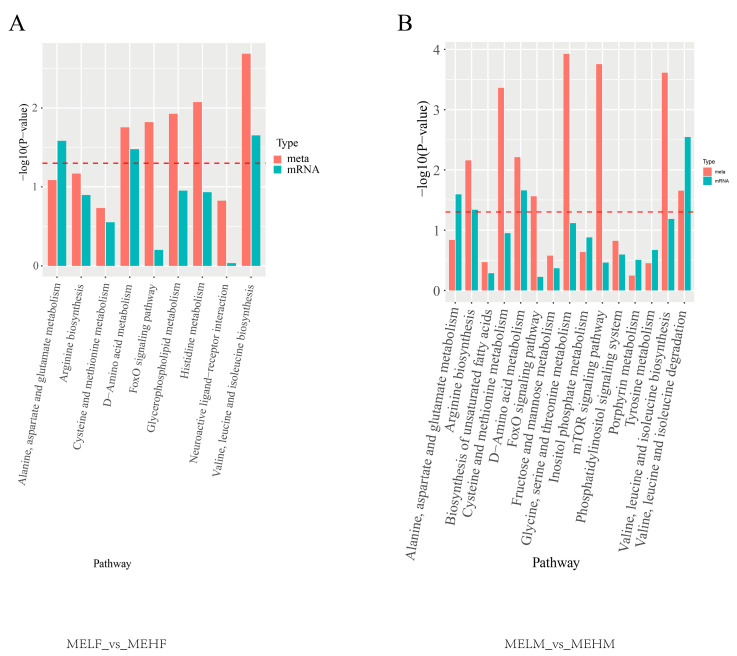
Co-enriched pathways from transcriptomic and metabolomic analyses in muscle tissue of large yellow croaker (*L. crocea*) with different muscle elasticity. The x-axis represents the metabolic pathways, while the y-axis represents the *p* values of the two omics enrichment analyses. The color represents different omics, the dotted line red line is position *p* = 0.05, and above the dotted line is *p* < 0.05. (**A**) Comparison between the female low muscle elasticity group and the female high muscle elasticity group. (**B**) Comparison between the male low muscle elasticity group and the male high muscle elasticity group.

Enrichment analysis based on DEMs and DEGs identified significant biochemical and signal transduction pathways. In the MELF_vs._MEHF group, the valine, leucine, and isoleucine biosynthesis pathway and the D-amino acid metabolism pathway were significantly enriched. In the MELM_vs._MEHM comparison group, arginine biosynthesis; valine, leucine, and isoleucine degradation; and D-amino acid metabolism pathways were significantly enriched (Figure 6).

#### 2.4.2. Correlation Analysis of Metabolites and Corresponding Transcripts

**Figure 7:**
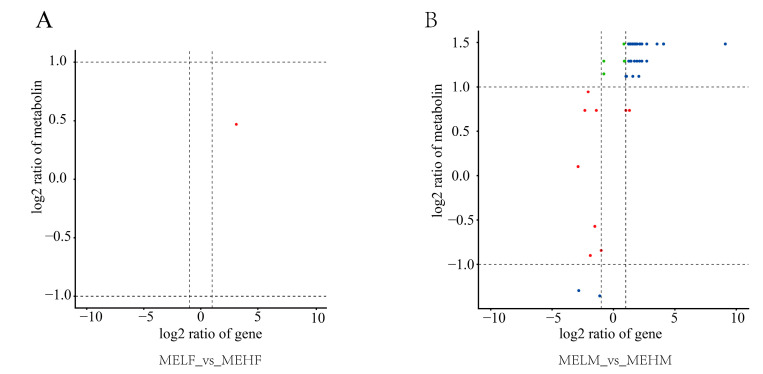
Correlation analysis of DEGs and DEMs in muscle tissue of large yellow croaker (*L. crocea*) with different muscle elasticity. The results show a Pearson correlation coefficient greater than 0.8, with transcriptome data plotted on the x-axis and metabolome data on the y-axis. (**A**) Comparison between the female low muscle elasticity group and the female high muscle elasticity group. (**B**) Comparison between the male low muscle elasticity group and the male high muscle elasticity group.

**Figure 8:**
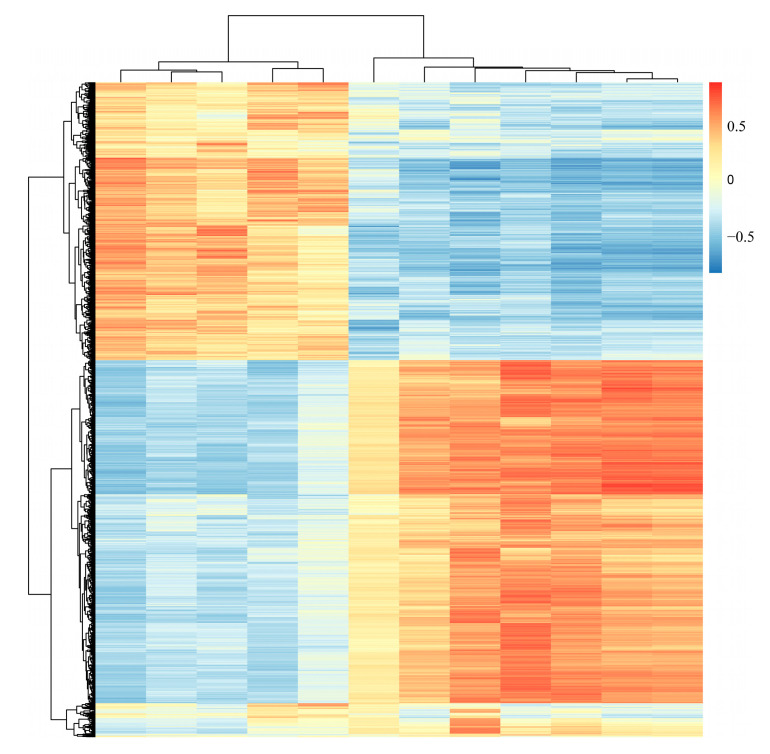
Heatmap showing the correlation analysis of DEGs and DEMs in muscle tissue of large yellow croaker (*L. crocea*) with different muscle elasticity. The horizontal represents genes, the vertical represents metabolite, and the color (blue to red) represents the correlation coefficient.

Results with a Pearson correlation coefficient greater than 0.8 were selected for the correlation analysis of DEGs and DEMs in the MELF_vs._MEHF and MELM_vs._MEHM comparison groups, as shown in Figure 7. In the MELF_vs._MEHF comparison, only one pair of DEGs and DEMs showed a correlation, making it impossible to generate a heatmap. In contrast, 47 pairs of DEGs and DEMs were associated in the MELM_vs._MEHM comparison group, and the heatmap is presented in Figure 8.

#### 2.4.3. Differential Expression Results of Metabolites and Related Transcripts

**Figure 9:**
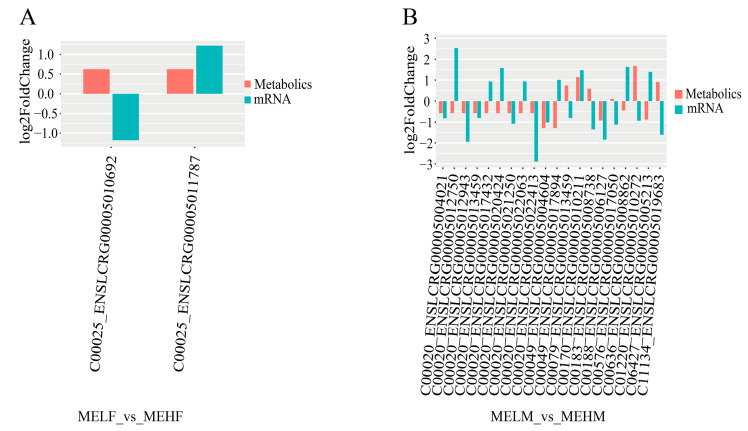
Differential expression results of DEMs and their associated DEGs in muscle tissue of large yellow croaker (*L. crocea*) with different muscle elasticity. The x-axis represents the names of related metabolites and transcripts, while the y-axis represents the fold change in differential expression. (**A**) Comparison between the female low muscle elasticity group and the female high muscle elasticity group. (**B**) Comparison between the male low muscle elasticity group and the male high muscle elasticity group.

Differential expression analysis revealed that in the MELF_vs._MEHF group, two DEMs and one DEG exhibited the same expression trend. In the MELM_vs._MEHM comparison group, 20 DEGs and 8 DEMs showed the same trend (Figure 9).

#### 2.4.4. Common Pathway Mapping of DEMs and DEGs 

To enhance the precision of the results, we mapped the DEMs and DEGs to the KEGG pathway database, identifying common pathways. In the MELF_vs._MEHF comparison group, the valine, leucine, and isoleucine biosynthesis pathway was significantly enriched, with DEMs including 2-oxoisovalerate and L-valine, and the DEGs encoding L-threonine ammonia-lyase (Table 4). In the MELM_vs._MEHM comparison group, arginine biosynthesis; arginine and proline metabolism; and valine, leucine, and isoleucine degradation pathways were significantly enriched, with DEMs including 4-guanidinobutanoate, L-aspartate, N-acetylornithine, and L-leucine, and DEGs including glutamine synthetase (*glul*), glutaminase (*gls*), spermidine synthase (*srm*), hydroxymethylglutaryl-CoA synthase (*hmgcs*), and acetoacetyl-CoA synthetase (*aacs*) (Table 4).

## 3. Materials and Methods

### 3.1. Experimental Fish

A total of 1200 one-year-old large yellow croakers, all with the same genetic background and an average body weight of 313.23 ± 90.99 g, were purchased from Shancheng farmers in Fuding City, Fujian Province, China. Before the experiment, the fish were acclimated for 48 h in indoor circular cement pools (diameter = 6 m, water depth = 1.5 m, 600 fish/pool) at the Fujian Fuding Research Center, East China Sea Fisheries Research Institute, Chinese Academy of Fishery Sciences. The temporary culture conditions were as follows: water temperature 27.0 ± 1.0 °C, salinity 25–26 parts per trillion (ppt), and dissolved oxygen (DO) > 6 mg/L. The fish were fed a commercial feed (the protein content is 48.21%, and the fat content is 8.11%) twice a day in the morning and evening to satiety, and half of the water was exchanged daily. This study has been approved by the Institutional Review Board of the Laboratory Animal Ethics Committee of the East China Sea Fisheries Research Institute (LAECECSFRI-2022-10-26-1).

### 3.2. Experimental Design

After acclimation, unhealthy, dead, and deformed fish were removed, leaving 1097 large yellow croakers. These fish were anesthetized using eugenol (MACKLIN, 20 mg/L) and the dorsal muscles above the lateral line were quickly excised. Muscle elasticity was measured using a texture analyzer (TMS-PRO, FTC, Sterling, VA, USA) following the local standards of Fujian Province. Each fish sample was measured six times. The muscle samples were then washed with phosphate-buffered saline (PBS), placed in enzyme-free frozen tubes, immediately frozen in liquid nitrogen, and stored at −80 °C.

The muscle elasticity values of the 1097 large yellow croakers were sorted from high to low. These continuous data follow a normal distribution (Figure S1). The highest and lowest muscle elasticity values at both ends of the distribution were selected, and from each group, 18 males and 18 females were randomly chosen, resulting in four groups: male high muscle elasticity (MEHM, 1.67 ± 0.091), female high muscle elasticity (MEHF, 1.57 ± 0.088), male low muscle elasticity (MELM, 0.83 ± 0.082), and female low muscle elasticity (MELF, 0.85 ± 0.10) (Table S1). Each group consisted of three replicates, with six muscle samples from large yellow croakers taken from each replicate for subsequent transcriptome and metabolomics analyses.

### 3.3. RNA Extraction, Transcriptome Sequencing, and DEGs Analysis

Total RNA was extracted using the Trizol reagent (Takara, Dalian, China) in strict accordance with the supplier’s operational specifications. The concentration and purity of the total RNA were measured using a NanoDrop 2000 spectrophotometer (Thermo Scientific, Waltham, MA, USA), and RNA integrity was measured using RNA-specific agarose electrophoresis or an Agilent 2100 Bioanalyzer with an RNA 6000 Nano kit 5067-1511(Agilent Technologies, Santa Clara, CA, USA). The quality of the RNA library was checked using an Agilent 2100 Bioanalyzer and an Agilent High Sensitivity DNA Kit (Agilent Technologies; 5067-4626). The total library concentration was determined using Pico green technology (Quantifluor-ST fluorometer, Promega, Madison, WI, USA, E6090; Quant-iT PicoGreen dsDNA Assay Kit, Invitrogen, Carlsbad, CA, USA, P7589). The effective library concentrations were quantified using quantitative real-time PCR (qPCR; StepOnePlus Real-Time PCR Systems, Thermo Fisher Scientific). Finally, 150 bp paired-end (PE150) mode sequencing was performed on an Illumina sequencer (Illumina Inc., San Diego, CA, USA). DESeq was used to analyze the differential gene expression, and the differential expression multiples and significant *p* values of each gene were calculated. The results of differential expression analysis were screened using the following conditions: differential expression multiples|log_2_FoldChange| > 1, significant *p* value < 0.05. Finally, two-way cluster analysis of the DEGs and samples from all comparison groups was performed using the Pheatmap package in R (version 4.3.2).

### 3.4. qRT-PCR for Data Validation

Each group was selected for 5 genes with a large difference in upregulation and downregulation. According to NCBI (https://www.ncbi.nlm.nih.gov/, accessed on 25 September 2024)-known large yellow croaker gene sequences, specific primers were designed using Primer-BLAST online (https://www.ncbi.nlm.nih.gov/tools/primer-blast/index.cgi?LINK_LOC=BlastHome, accessed on 25 September 2024) (Table S2). According to the instructions of the fluorescent quantitative kit, the Quant Studio 6 FLEX fluorescent quantitative PCR instrument was used, and the fluorescent quantitative response was performed with the *β*-actin primer and the above primers. The reaction system was as follows: 2 × Ultra Sybr MixTure of 12.5 μL, and the 0.5 μL Forward Primer (10 μmol·L^−1^), 0.5 μL of Reverse Primer (10 μmol·L^−1^), 1 μL Template cDNA, and 10.5 μL ddH_2_O. The two-step PCR amplification standard program was adopted; the parameters were as follows: prestigious (95 °C, 10 min), degeneration (95 °C, 15 s), annealing extension (60 °C, 1 min), 40 cycles. The experimental data obtained were processed using the 2^−ΔΔCT^ method to calculate the relative expression of each gene in each group in the muscle tissue, DEGs (*p* < 0.05) and DEMs (VIP > 1, *p* < 0.05).

### 3.5. Metabolomic Analysis

For metabolite extraction, samples were thawed slowly at 4 °C and an appropriate amount of sample was added to a pre-cooled methanol/acetonitrile/water solution (2:2:1, *v/v*). The mixture was vortexed, sonicated at a low temperature for 30 min, left to stand at −20 °C for 10 min, centrifuged at 14,000× *g* at 4 °C for 20 min, and the supernatant was retained and vacuum-dried. For mass spectrometry analysis, the dried sample was re-dissolved in 100 μL of acetonitrile/water solution (1:1, *v/v*), vortexed, centrifuged at 14,000× *g* at 4 °C for 15 min, and the supernatant was used for further analysis.

Metabolites were separated using a Vanquish LC ultra-high performance liquid chromatography (UHPLC) system with a hydrophilic interaction liquid chromatography (HILIC) column (Thermo Fisher Scientific, Waltham, MA, USA). Mass spectrometry analysis was performed using a Q Exactive series mass spectrometer (Thermo Fisher Scientific, Waltham, MA, USA), utilizing electrospray ionization (ESI) in both positive and negative ion modes. Metabolites were identified using a combination of local databases and public databases, such as the Human Metabolome Database (HMDB, http://www.hmdb.ca, accessed on 23 July 2023), Metlin (https://metlin.scripps.edu, accessed on 23 July 2023), MassBank (http://www.massbank.jp, accessed on 23 July 2023), and mzCloud (https://www.mzcloud.org, accessed on 23 July 2023). Metabolites were identified by matching retention times, molecular masses (with a mass error < 10 ppm), fragmentation spectra, and collision energies with database entries, followed by manual verification. Significant differentially expressed metabolites (DEMs) were identified using orthogonal partial least squares discriminant analysis (OPLS-DA) with a Variable Importance in Projection (VIP) score > 1 and *p* < 0.05 as screening criteria.

### 3.6. Metabolome and Transcriptome Association Analysis

The relationships between DEMs and transcripts associated with enzymes were identified using the Kyoto Encyclopedia of Genes and Genomes (KEGG) database (https://www.kegg.jp/kegg/ko.html, accessed on 5 September 2023). Transcript information, including K numbers, gene names, EC numbers, and associated metabolic pathways, was retrieved from the KEGG homologous gene database. Transcripts and DEMs were then aligned and their common pathways were identified. Bar charts were drawn to illustrate the significance of co-enriched pathways based on individual omics data from the DEMs and DEGs. For higher precision, both DEMs and all transcripts were mapped to the KEGG pathway database to identify common pathways for DEGs (*p* < 0.05) and DEMs (VIP > 1, *p* < 0.05).

## 4. Discussion

Fish muscle quality is influenced by both genetics and the environment [22,23]. The type and distribution of muscle fibers vary significantly among different fish species, leading to diverse meat characteristics [24]. Environmental factors such as the water temperature, DO levels, feed composition, and feeding practices also play crucial roles in determining fish muscle quality [25,26,27]. Among various evaluation methods for fish muscle quality, sensory evaluation is a direct, but subjective, approach, often lacking consistency and accuracy. Muscle elasticity, a key indicator of fish muscle quality, is crucial to assess the freshness and texture of fish. To date, most studies have focused on the impact of environmental factors on muscle elasticity [28,29], while the molecular mechanisms underlying its formation remain largely unexplored. Therefore, this study systematically analyzed the molecular regulatory mechanisms of muscle elasticity in large yellow croaker through a combined multi-omics approach to provide a scientific basis to optimize culture conditions and to breed new species variants.

D-amino acids have traditionally been considered less important in animals because they are non-protein amino acids. However, recent studies have shown that D-amino acids also play important roles in biological processes such as neurotransmission, and regulation of antioxidants and metabolism [30,31]. Notably, the D-amino acid metabolic pathway was significantly enriched in both the MELF_vs._MEHF and MELM_vs._MEHM comparison groups in this study, suggesting that D-amino acids in certain metabolic pathways might influence muscle growth and function. It has been shown that D-amino acid oxidase converts D-amino acids into corresponding keto acids and ammonia, which can further participate in energy metabolism and anabolism, potentially affecting the metabolic activity of muscle cells [32]. Moreover, the role of D-serine in neurotransmission might indirectly affect fish growth and muscle quality by affecting their behavior and stress responses [33]. Therefore, D-amino acid metabolism might play an important role in regulating the muscle elasticity of large yellow croaker, although the specific mechanisms require further experimental validation.

In the MELF_vs._MEHF comparison group, 2-oxoisovalerate and L-valine were significantly enriched in the biosynthetic pathways of valine, leucine, and isoleucine. Valine, leucine, and isoleucine, collectively known as branched-chain amino acids (BCAAs), serve not only as substrates for protein synthesis, but also as vital regulators of growth, physiology, and metabolic processes in fish [34]. Valine is involved in the transamination of BCAAs, producing 2-oxoisovalerate, with skeletal muscle being the primary site for this process. Studies have shown that BCAAs are key to the energy metabolism of muscle tissues in various fish species and positively affect muscle quality [35,36]. For example, adding valine to the diet has been shown to improve the flavor and antioxidant capacity of grass carp [37]. Similarly, leucine supplementation enhanced muscle fiber growth and development in blunt snout bream (*Megalobrama amblycephala*), leading to improved muscle quality [37]. These findings underscore the importance of BCAAs in regulating muscle elasticity in fish.

In contrast, the MELM_vs._MEHM comparison group showed significant enrichment in the arginine biosynthesis pathway, with *glul* and *gls* as significant DEGs, and 4-guanidinobutanoate as a significant DEM. Arginine plays a key role in fish growth and development and physiological functions, including promoting fish growth, enhancing immune function, improving reproductive function, regulating body metabolism, and improving feed utilization [38,39,40,41,42]. For example, arginine has been shown to improve the lipid content, hardness, and hydrolyzed amino acid content of grass carp muscle; increase the proportion of hyperplastic fibers; and decrease the proportion of hypertrophic fibers, thereby improving muscle quality [43]. In other fish species, such as Nile tilapia (*Oreochromis niloticus*) [44] and sturgeon (genus *Acipenser*) [45], arginine contributes to skeletal muscle development. Glutamine, a free amino acid found in high concentrations in plasma and skeletal muscle, is involved in various biosynthetic and metabolic processes [46]. For example, glutamine synthetase (encoded by *glul*) has been associated with amino acid metabolism in muscle tissue, with its catalysis generating *gls*, which is essential for muscle growth and repair in *Cyprinus carpio* [47]. The upregulation of *glul* and *gls* expression in the MEHM group suggests that these genes might influence muscle growth and development in large yellow croaker through amino acid metabolic pathways, thereby affecting muscle elasticity. The metabolite 4-guanidinobutanoate, derived from arginine metabolism, is involved in various metabolic activities and has high physiological activity [48]. This suggests that arginine metabolism in large yellow croaker might influence 4-guanidinobutanoate synthesis, subsequently affecting muscle elasticity.

Furthermore, the arginine and proline metabolism pathways, as well as the valine, leucine, and isoleucine degradation pathways, were significantly enriched in the MELM_vs._MEHM comparison group. The significant upregulation of genes such as *srm*, *hmgcs*, and *aacs,* along with metabolites like L-aspartate, N-acetylornithine, and L-leucine, in the MEHM group highlights their roles in muscle elasticity. Proline, an important amino acid in muscle, contributes to the strength and toughness of muscle fibers because of its rigid structure and plays a role in regulating connective tissue, thereby influencing muscle quality [49,50,51]. The connective tissue of muscle is a key determinant of tenderness and aging, with studies showing that changes in the connective tissue of brittle grass carp result in meat quality differences compared with those in ordinary grass carp [52]. Based on these findings, proline might influence muscle elasticity in large yellow croakers through amino acid metabolism. Moreover, studies have demonstrated that L-aspartate supplementation in pig diets improves meat quality [53], while low-protein diets reduce N-acetylornithine in pig muscle, highlighting the relationship between protein and this amino acid [16]. This suggests that L-aspartate and N-acetylornithine play critical roles in muscle elasticity in large yellow croaker. Spermidine synthase, encoded by *srm*, is involved in polyamine biosynthesis and has an essential role in muscle growth and development [54,55,56,57]. Alterations in spermidine metabolic levels can lead to changes in skeletal muscle [58]. For example, spermidine inhibited skeletal muscle cell senescence in a rat model, playing a crucial role in delaying muscle atrophy [57]. Additionally, spermidine’s effects on free radical metabolism in mouse skeletal muscle showed that it can enhance intracellular free radical scavenging enzyme activity, improving metabolism and resistance to muscle cell membrane damage [59]. Thus, spermidine synthase might affect muscle quality through amino acid metabolism. *Hmgcs* exists in two forms: *hmgcs1* encodes a protein involved in cholesterol and isoprenoid biosynthesis [60], while *hmgcs2* encodes a protein that participates in ketone body production [61,62]. Cholesterol biosynthesis is essential for cell membrane structure and function [63], and ketone body production plays a key role in regulating energy supply and metabolism [64], thereby influencing the metabolic activity and energy supply of muscle cells. Acetoacetyl-CoA synthetase (encoded by *aacs*) is primarily involved in acetoacetate biosynthesis and has significant effects on fatty acid and lipid metabolism [65,66], potentially affecting muscle texture. These results further highlight the importance of amino acid metabolism in regulating muscle quality in large yellow croakers.

However, several potential limitations may affect the results of this study. Notably, inbreeding may impact muscle elasticity, as the reduction in genetic diversity can lead to phenotypic homogenization and a decline in flavor and quality [67,68]. To address this concern, future research should incorporate assessments of genetic diversity and explore the impact of breeding strategies on muscle elasticity traits. Additionally, integrating other parameters, such as environmental factors, dietary influences, and gut microbiota composition, will help elucidate the regulatory mechanisms underlying muscle elasticity in large yellow croakers, providing a theoretical foundation for the future cultivation of high-quality large yellow croakers.

## 5. Conclusions

This study investigated the molecular regulation mechanisms of muscle elasticity traits in large yellow croaker through transcriptomics and metabolomics analyses. In the MELF_vs._MEHF comparison, the valine, leucine, and isoleucine biosynthesis pathways was significantly enriched. Meanwhile, in the MELM_vs._MEHM comparison, significant enrichment was observed in the arginine biosynthesis, arginine and proline metabolism, and valine, leucine, and isoleucine degradation pathways. Specifically, in females, 2-oxoisovalerate and L-valine were markedly upregulated in the high elasticity group. In males, 4-guanidinobutanoate, L-aspartate, N-acetylornithine, L-leucine, and key genes such as *glul*, *gls*, *srm*, *hmgcs*, and *aacs* were significantly upregulated in the high elasticity group. These findings highlight the pivotal role of amino acid metabolism in regulating muscle quality in large yellow croaker, providing valuable insights into the molecular mechanisms underlying muscle elasticity. This research lays a theoretical foundation for the cultivation of high-quality large yellow croakers.

## Figures and Tables

**Table 1:** Quality metrics of raw and clean reads for muscle tissue of large yellow croaker (*L. crocea*) transcriptomic analysis with different muscle elasticity.

Sample	Raw_Reads	Clean_Reads	Q20 (%)	Q30 (%)
MEHF_1	6,264,928,694	6,106,530,964	96.78	94.26
MEHF_2	6,026,724,080	5,882,453,846	96.86	94.41
MEHF_3	6,727,502,396	6,568,158,095	96.82	94.33
MEHM_1	6,380,928,706	6,213,714,638	96.65	94.03
MEHM_2	6,895,087,330	6,737,222,132	96.9	94.48
MEHM_3	5,837,551,582	5,708,983,823	97	94.64
MELF_1	6,124,735,462	5,968,151,868	96.82	94.34
MELF_2	6,391,422,300	6,236,534,936	96.79	94.28
MELF_3	6,701,982,188	6,562,916,923	96.98	94.6
MELM_1	8,782,453,544	8,561,838,358	96.87	94.41
MELM_2	7,848,064,638	7,665,420,808	96.88	94.45
MELM_3	6,979,125,474	6,816,427,089	96.82	94.32

MEHM, male high muscle elasticity; MEHF, female high muscle elasticity; MELM, male low muscle elasticity; MELF, female low muscle elasticity.

**Table 2:** Mapping statistics for muscle tissue of large yellow croaker (*L. crocea*) transcriptomic analysis with different muscle elasticity.

Sample Name	Total Mapped	Multiple Mapped	Unique Mapped
MEHF_1	39,146,176 (96.35%)	3,201,856 (8.18%)	35,944,320 (91.82%)
MEHF_2	37,588,215 (96.11%)	2,970,506 (7.90%)	34,617,709 (92.10%)
MEHF_3	42,105,944 (96.52%)	3,467,629 (8.24%)	38,638,315 (91.76%)
MEHM_1	39,531,273 (95.67%)	3,110,858 (7.87%)	36,420,415 (92.13%)
MEHM_2	43,062,855 (96.21%)	3,700,673 (8.59%)	39,362,182 (91.41%)
MEHM_3	36,470,763 (96.01%)	2,912,774 (7.99%)	33,557,989 (92.01%)
MELF_1	38,083,393 (95.90%)	3,415,752 (8.97%)	34,667,641 (91.03%)
MELF_2	39,950,939 (96.32%)	3,171,414 (7.94%)	36,779,525 (92.06%)
MELF_3	41,907,924 (96.04%)	3,339,504 (7.97%)	38,568,420 (92.03%)
MELM_1	54,967,112 (96.51%)	5,563,230 (10.12%)	49,403,882 (89.88%)
MELM_2	49,157,327 (96.48%)	4,488,611 (9.13%)	44,668,716 (90.87%)
MELM_3	43,719,356 (96.53%)	3,565,020 (8.15%)	40,154,336 (91.85%)

**Table 3:** Statistical analysis of differential metabolites in muscle tissue of large yellow croaker (*L. crocea*) with different muscle elasticity.

Indicators		MELF_vs._MELM	MELF_vs_MEHF	MELM_vs. MEHM	MEHF_vs. MEHM
All metabolites	Pos	479			
	Neg	490			
Differential metabolites	Pos	16	27	28	7
	Neg	9	20	29	8
Upregulated	Pos	9	9	9	2
	Neg	3	8	14	4
Downregulated	Pos	7	18	17	5
	Neg	6	12	15	4

DEM, differentially expressed metabolite; Pos, positive; Neg, negative.

**Table 4:** KEGG pathways from the combined transcriptomic and metabolomic analyses of muscle tissue in large yellow croaker (*L. crocea*) with different muscle elasticity, along with associated DEGs and DEMs.

Group	Pathway	DEMs	DEGs
MELF_vs._MEHF	Valine, leucine and isoleucine biosynthesis	2-Oxoisovalerate, L-Valine	L-threonine ammonia-lyase
MELM_vs._MEHM	Arginine biosynthesis	4-Guanidinobutanoate	*glul*, *gls*
	Arginine and proline metabolism	L-Aspartate, N-Acetylornithine	*srm*
	Valine, leucine and isoleucine degradation	L-Leucine	*hmgcs*, *aacs*

DEG, differentially expressed gene.

## Data Availability

The RNA-seq data supporting the findings of this study have been deposited in the NCBI Sequence Read Archive (SRA) under accession number (PRJNA1148757).

## Supplementary Materials

The following supporting information can be downloaded at: https://www.mdpi.com/article/10.3390/ijms252010924/s1.

## References

[1] Dou X., Wang Y.-Q., Wu Y.-Y., Hu X., Yang S.-L., Li C.-S., Cen J.-W. (2020). Analysis and evaluation of nutritional components in liver of large yellow croaker (*Pseudosciaena crocea*). Cyta. J. Food..

[2] Ding H.-Y., Sun X.-J., Sheng X.-F., Zhao Y.-F., Shang D.-R., Zhai Y.-X. (2016). Comparison and analysis of the nutritional composition in muscle of some cultured fresh-water and marine-cultured fishes. Food Sci. Technol..

[3] Zhang H., Wang J., Jing Y. (2024). *Larimichthys crocea* (large yellow croaker): A bibliometric study. Heliyon.

[4] Ma J.-K. (2021). Germplasm Resources and Population Genetics Analysis of *Larimichthys crocea* in the Offshore of China. Master’s Thesis.

[5] Yuan J., Zhuang X., Wu L., Lin H., Li Y., Wu L., Yao J., Liu J., Ding S. (2024). Assessing the population genetic structure of yellow croaker in China: Insights into the ecological and genetic consequences of artificial breeding on natural populations. Aquaculture.

[6] Lin S., Wang S., Li J. (2002). The Feeding Experimemt of Larvae *Pseudosciaena Crocea* with Formula Feeds. J. Fujian Agr. For. Univ. (Nat. Sci. Ed.).

[7] Hong W.-S., Zhang Q.-Y. (2002). Artificial propagation and breeding of marine fish in China. Chin. J. Oceanol. Limn..

[8] Imamura S., Suzuki M., Okazaki E., Murata Y., Kimura M., Kimiya T., Hiraoka Y. (2012). Prevention of Thaw-Rigor During Frozen Storage of Bigeye Tuna Thunnus Obesus and Meat Quality Evaluation. Fish. Sci..

[9] Feng L., Peng Y., Wu P., Hu K., Jiang W., Liu Y., Jiang J., Li S., Zhou X. (2013). Threonine Affects Intestinal Function, Protein Synthesis and Gene Expression of TOR in Jian Carp (*Cyprinus carpio var.* Jian). PLoS ONE.

[10] Liao Y., Ren M., Liu B., Sun S., Cui H., Xie J., Zhou Q., Pan L., Chen R., Ge X. (2014). Dietary Methionine Requirement of Juvenile Blunt Snout Bream (*Megalobrama amblycephala*) at a Constant Dietary Cystine Level. Aquacult. Nutr..

[11] Zhao Z., Song F., Wang L., Luo L., Wang C., Li J., Du X., Xu Q. (2018). Effects of Gln and Its Precursors on Muscular Approximate Composition, Amino Acid Composition and AKP Activities in Songpu Mirror Carp Cyprinus Carpio Songpu. J. Dalian Ocean Univ..

[12] Tie H., Wu P., Jiang W., Liu Y., Kuang S., Zeng Y., Jiang J., Tang L., Zhou S., Feng L. (2019). Dietary Nucleotides Supplementation Affect the Physicochemical Properties, Amino Acid and Fatty Acid Constituents, Apoptosis and Antioxidant Mechanisms in Grass Carp (*Ctenopharyngodon idellus*) muscle. Aquaculture.

[13] Fuentes A., Fernández-Segovia I., Serra J.A., Barat J.M. (2010). Comparison of Wild and Cultured Sea Bass (*Dicentrarchus labrax*) Quality. Food Chem..

[14] Lenas D., Chatziantoniou S., Nathanailides C., Triantafillou D. (2011). Comparison of Wild and Farmed Sea Bass (*Dicentrarchus labrax L*) Lipid Quality. Procedia Food Sci..

[15] Guan W., Zhu Y., Chen Z. (2008). Muscle Quality in Fish Related to Characteristics of Muscular Fibers. Fish. Sci..

[16] Hurling R., Rodell J.B., Hunt H.D. (1996). Fiber diameter and fish texture. J. Texture Stud..

[17] Liang X., Hu L., Dong Y., Wu X., Qin Y., Zheng Y., Shi D., Xue M. (2017). Substitution of Fish Meal by Fermented Soybean Meal Affects the Growth Performance and Flesh Quality of Japanese Seabass (*Lateolabrax japonicus*). Anim. Feed Sci. Tech..

[18] Bai Z., Liu M., Li S., Lin H., Lin L., Li R., Chen Z., Chen X. (2019). Effect of Dietary Threonine Supplement on the Fillet Quality and Cathepsin B/L Level of Jian carp (*Cyprinus carpio* var Jian). Food. Fer. Ind..

[19] Yu E., Xie J., Wang G., Yu D., Gong W., Li Z., Wang H., Xia Y., Wei N. (2014). Gene Expression Profiling of Grass Carp (*Ctenopharyngodon idellus*) and Crisp Grass Carp. Int. J. Genom..

[20] Wang L., Xiong J., Xu C., Qin C., Zhang Y., Yang L., Zhi S., Feng J., Nie G. (2024). Comparison of Muscle Nutritional Composition, Texture Quality, Carotenoid Metabolites and Transcriptome to underling Muscle Quality Difference between Wild-Caught and Pond-Cultured Yellow River Carp (*Cyprinus carpio haematopterus*). Aquaculture.

[21] Wei Z., Zhou H., Zhang Y., Zhang Q., Zhang W., Mai K. (2018). Integrative Analysis of Transcriptomics and Metabolomics Profiling on Flesh Quality of Large Yellow Croaker *Larimichthys crocea* Fed a Diet with Hydroxyproline Supplementation. Brit. J. Nutr..

[22] Cai L., Tong F., Tang T., Ao Z., Wei Z., Yang F., Shu Y., Liu S., Mai K. (2021). Comparative Evaluation of Nutritional Value and Flavor Quality of Muscle in Triploid and Diploid Common Carp: Application of Genetic Improvement in Fish Quality. Aquaculture.

[23] Wang L., Wang L., Liu C., Ma F., Huang J., Jin Z., Zhang L., Feng D., Zhang M., Yu M. (2024). Multi-Omics Reveals the Molecular Mechanism of Muscle Quality Changes in Common Carp (*Cyprinus carpio*) under Two Aquaculture Systems. Comp. Biochem. Phys. D.

[24] Valente L.M., Moutou K.A., Conceicao L.E., Engrola S., Fernandes J.M., Johnston I.A. (2013). What Determines Growth Potential and Juvenile Quality of Farmed Fish Species?. Rev. Aquacult..

[25] Johnston I.A., Li X., Vieira V.L., Nickell D., Dingwall A., Alderson R., Campbell P., Bickerdike R. (2006). Muscle and Flesh Quality Traits in Wild and Farmed Atlantic Salmon. Aquaculture.

[26] Gao X., Zhai H., Peng Z., Yu J., Yan L., Wang W., Han Y. (2023). Comparison of Nutritional Quality, Flesh Quality, Muscle Cellularity, and Expression of Muscle Growth-Related Genes Between Wild and Recirculating Aquaculture System (RAS)-Farmed Black Rockfish (*Sebastes Schlegelii*). Aquacult. Int..

[27] Wei Z., Ma J., Pan X., Mu H., Li J., Shentu J., Zhang W., Mai K. (2016). Dietary Hydroxyproline Improves the Growth and Muscle Quality of Large Yellow Croaker *Larimichthys crocea*. Aquaculture.

[28] Lu G., Yao Z., Lai Q., Gao P., Zhou K., Zhu H., Liu Y., Sun Z. (2022). Growth Performance, Blood Parameters, and Texture Characteristics of Juvenile Largemouth Bass (*Micropterus salmoides*) Exposed to Highly Saline-Alkaline Water. Prog. Fish. Sci..

[29] Zhang B., Dong Z., Kang J., Wang B., Cai W., Shi B., Zhang X. (2024). Optimizing Full Plant Protein Feed through Nutritional and Un-nutritional Methods on the Compositions and Texture Properties of Largemouth Bass Micropterus Salmoides Muscle. Acta Hydrobiol. Sin..

[30] Genchi G. (2017). An Overview on D-Amino Acids. Amino Acids..

[31] Seckler J.M., Lewis S.J. (2020). Advances in D-Amino Acids in Neurological Research. Int. J. Mol. Sci..

[32] Ryuichi K., Yosihiro Y. (1992). D-Amino-Acid Oxidase and Its Physiological Function. Int. J. Biochem..

[33] Wolosker H., Blackshaw S., Snyder S.H. (1999). Serine Racemase: A Glial Enzyme Synthesizing D-Serine to Regulate Glutamate-N-Methyl-D-Aspartate Neurotransmission. Proc. Natl. Acad. Sci. USA.

[34] Ahmad I., Ahmed I., Fatma S., Peres H. (2021). Role of Branched-Chain Amino Acids on Growth, Physiology and Metabolism of Different Fish Species: A Review. Aquacult. Nutr..

[35] Li H., An X., Bao L., Li Y., Pan Y., He J., Liu L., Zhu X., Zhang J., Cheng J. (2020). MiR-125a-3p-KLF15-BCAA Regulates the Skeletal Muscle Branched-Chain Amino Acid Metabolism in Nile tilapia (*Oreochromis niloticus*) during Starvation. Front. Genet..

[36] Huang W., Zhang Z., Shi Y., Zhang G., Zhang H., Lu G., Xie Y., Luo Z. (2014). Analysis and Evaluation of Nutritional Components in Muscle of Cultured Synechogobius Ommaturus. Chin. J. Anim. Nutr..

[37] Luo J., Feng L., Jiang W., Liu Y., Wu P., Jiang J., Kuang S., Tang L., Tang W., Zhang Y. (2017). Physical and Flavor Characteristics, Fatty Acid Profile, Antioxidant Status and Nrf2-Dependent Antioxidant Enzyme Gene Expression Changes in Young Grass Carp (*Ctenopharyngodon idella*) fillets fed dietary valine. PLoS ONE.

[38] Li P., Hou D., Zhao H., Peng K., Chen B., Guo H., Cao J. (2022). Effects of Dietary Arginine Levels on Intestinal Morphology, Digestive Enzyme Activity, Antioxidant Capacity and Intestinal Flora of Hybrid Snakehead (*Channa maculata*♀× *Channa argus*♂). Aquacult. Rep..

[39] Wang Y., Wang C.A., Liu S., Zhang S., Lu S., Liu H., Han S., Jiang H., Zhang Y. (2022). Effects of Dietary Arginine on Growth Performance, Digestion, Absorption Ability, Antioxidant Capability, Gene Expression of Intestinal Protein Synthesis, and Inflammation-Related Genes of Triploid Juvenile Oncorhynchus mykiss Fed a Low-Fishmeal Diet. Aquacult. Nutr..

[40] Wu Y., Dai Y., Xiao K., Wang X., Wang M., Huang Y., Liu W. (2022). Effects of Different Dietary Ratio Lysine and Arginine on Growth, Muscle Fiber Development and Meat Quality of *Megalobrama amblycephala*. Aquacult. Rep..

[41] Mao X., Wang Y., Zhang T., Ma J., Zhao J., Xu D. (2024). Dietary Arginine Regulates the Growth Performance, Antioxidant Capacity, and Immune Response in Culter Alburnus. Fish Physiol. Biochem..

[42] Xiong Y., Wang X., Sun R., Jiang Y., He Z., Chen J., Mei J. (2024). Administration of Arginine Vasotocin and Modified Isotocin Improve Artificial Propagation and Post-Spawning Survival of Female Yellow Catfish. Aquaculture.

[43] Ma Y., Zhou X., Wu P., Jiang W., Liu Y., Ren H., Zhang R., Li S., Tang L., Feng L. (2024). New Sight in Arginine-Improved Flesh Quality: Role of MRFs, Cyclins, and WNT Signaling in Grass Carp (*Ctenopharyngodon idellus*). Aquaculture.

[44] Neu D., Boscolo W., Zaminhan M., Almeida F., Sary C., Furuya W. (2016). Growth Performance, Biochemical Responses, and Skeletal Muscle Development of Juvenile Nile Tilapia, Oreochromis Niloticus, Fed with Increasing Levels of Arginine. J. World Aquacult. Soc..

[45] Yang S., Liu Z., Yan Z., Zhao Z., Zhang C., Gong Q., Huang X. (2021). Improvement of Skeletal Muscle Growth by GH/IGF Growth-Axis Contributes to Growth Performance in Commercial Fleshy Sturgeon. Aquaculture.

[46] Hamre K., Bjørnevik M., Espe M., Conceição L.E., Johansen J., Silva J., Hillestad M., Prabhu A.J., Taylor J.F., Tocher D.R. (2020). Dietary Micronutrient Composition Affects Fillet Texture and Muscle Cell Size in Atlantic Salmon (*Salmo salar*). Aquacult. Nutr..

[47] Wu Y., Xiao P., Sha H., Luo X., Zou G., Liang H. (2024). Transcriptome Analysis Reveals the Potential Key Genes in Nutritional Deposition in the Common Carp (*Cyprinus carpio*). Animals.

[48] Hu Z., Liu X. (2023). Integration of Transcriptomics and Non-Targeted Metabolomics Reveals the Underlying Mechanism of Skeletal Muscle Development in Duck during Embryonic Stage. Int. J. Mol. Sci..

[49] Chen S., Zeng M., Dong S. (2004). Progress in The Study of Collagen and Active Peptide of Fisheries. Fish. Sci..

[50] Li X., Zhao W., Sha L. (2008). Effects of the Characteristics of Muscle Collagen on Tenderness. Meat Res..

[51] Mccormick R.-J. (1999). Extracellular modifications to muscle collagen: Implications for meat quality. Poultry Sci..

[52] Wu F., Lin W., Li L., Yang X., Hao S., Yang S., Wei Y. (2015). Changes in Muscle Collagen Content, Mineral Contents and Fatty Acid Composition of Grass Carp during Crisping Process. Food Sci..

[53] Zeng D. (2023). Whole-Genome Resequencing and Transcriptome Analysis to Identify Gene and SNP Related to Growth in Pelodiscus Sinensis. Ph.D. Thesis.

[54] Persson L., Khomutov A.R., Khomutov R.M. (1989). Feedback Regulation of S-Adenosylmethionine Decarboxylase Synthesis. Biochem. J..

[55] Cepero M., Cubria J.C., Reguera R., Balana-Fouce R., Ordonez C., Ordonez D. (1998). Plasma and Muscle Polyamine Levels in Aerobically Exercised Rrats Treated with Salbutamol. J. Pharm. Pharmcol..

[56] Turchanowa, Rogozkin, Milovic, Feldkoren, Caspary (2000). Influence of Physical Exercise on Polyamine Synthesis in the Rat Skeletal Muscle. Eur. J. Clin. Investig..

[57] Li H., Chen Z., Lv X. (2024). Recent Progress on Spermidine Alleviating Cell Senescence and Agingrelated Diseases. Curr. Biotechnol..

[58] Zhang M., Lin Y., Wang Z., Li S., Li R., Chen M., Li Y., Yin P., Zhang L., Tang P. (2023). Analysis on The Mechanism of Skeletal Muscle Atrophy in Opg Gene Knockout Mice Based on Metabolomics. Med. J. Chin. People’s Lib. Army.

[59] He E., Tang L., Guo Y. (2014). Influence of Spermidine on Free Radical Metabolism in Skeletal Muscle and Its Anti-fatigue Effect in Mice. Food Sci..

[60] Zuo J., Ma S. (2024). Bioinformatics Analysis and Validation of Differentially Expressed Genes and Small Molecule Drug Prediction in Proliferative Scar. Chin. J. Tissue Eng. Res..

[61] Asif S., Kim R.Y., Fatica T., Sim J., Zhao X., Oh Y., Denoncourt A., Cheung A.C., Downey M., Mulvihill E.E. (2022). Hmgcs2-Mediated Ketogenesis Modulates High-Fat Diet-Induced Hepatosteatosis. Mol. Metab..

[62] Liu Y., Ma L., Fu P. (2023). Ketone Body Metabolism and Renal Diseases. J. Sichuan Univ. (Med. Sci.).

[63] Yeagle P.L. (1985). Cholesterol and the Cell Membrane. BBA-Rev. Biomembr..

[64] Cahill G.F. (2006). Fuel Metabolism in Starvation. Annu. Rev. Nutr..

[65] Tsuda M., Fukushima A., Matsumoto J., Takada S., Kakutani N., Nambu H., Yamanashi K., Furihata T., Yokota T., Okita K. (2018). Protein Acetylation in Skeletal Muscle Mitochondria is Involved in Impaired Fatty Acid Oxidation and Exercise Intolerance in Heart Failure. J. Cachexia Sarcopeni.

[66] Mooli R.G.R., Ramakrishnan S.K. (2022). Emerging Role of Hepatic Ketogenesis in Fatty Liver Disease. Front. Physiol..

[67] Östergren J., Palm S., Gilbey J., Spong G., Dannewitz J., Königsson H., Person J., Vasemägi A. (2021). A century of genetic homogenization in Baltic salmon—Evidence from archival DNA. Proc. R. Soc. B.

[68] Paul K., D’Ambrosio J., Phocas F. (2022). Temporal and region-specific variations in genome-wide inbreeding effects on female size and reproduction traits of rainbow trout. Evol. Appl..

