# Supplementary material for: Unveiling the Molecular Mechanisms Regulating Muscle Elasticity in the Large Yellow Croaker: Insights from Transcriptomics and Metabolomics
Source: Int J Mol Sci. 2024 Oct 11;25(20):10924. doi: 10.3390/ijms252010924 (PMC11507341; doi:10.3390/ijms252010924)
Supplement: Supplementary file 1 [file ijms-25-10924-s001.zip › Figure S1.pdf]

A

B

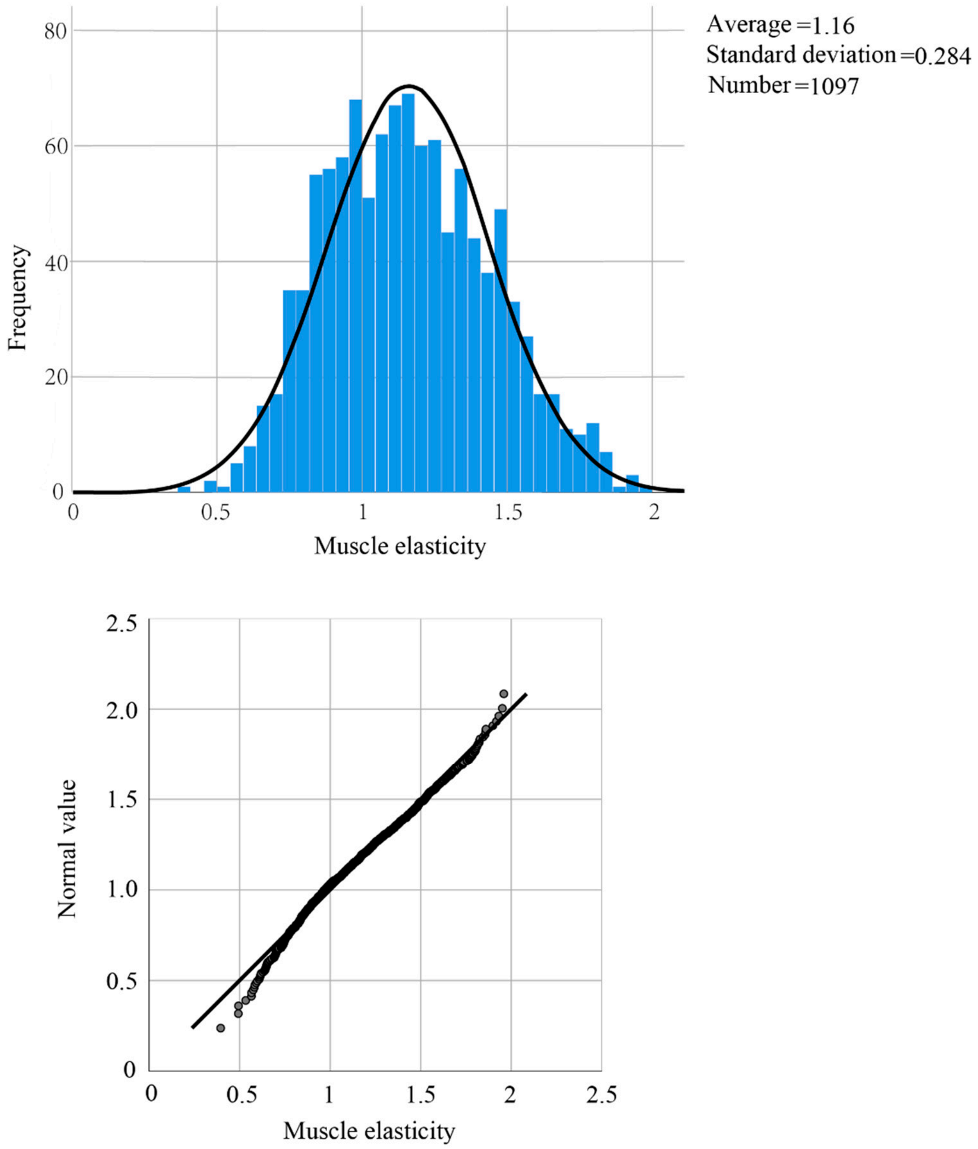

Figure S1. Normality test of muscle elasticity values in large yellow croaker. (**A**) Normal distribution histogram. (**B**) Normal distribution Q-Q plot.
