# Supplementary material for: Unveiling the Molecular Mechanisms Regulating Muscle Elasticity in the Large Yellow Croaker: Insights from Transcriptomics and Metabolomics
Source: Int J Mol Sci. 2024 Oct 11;25(20):10924. doi: 10.3390/ijms252010924 (PMC11507341; doi:10.3390/ijms252010924)
Supplement: Supplementary file 1 [file ijms-25-10924-s001.zip › Table S1.pdf]

Table S1. Muscle elasticity statistics of four groups of large yellow croaker (*L. crocea*).

High Muscle Elasticity			Low Muscle Elasticity		
**Number**	**Elasticity Values**	**Sex**	**Number**	**Elasticity Values**	**Sex**
91	1.803171317	F	640	0.680472267	F
148	1.690625433	F	89	0.6992389	F
259	1.673932767	F	842	0.71183905	F
44	1.626770133	F	515	0.775805383	F
238	1.616696233	F	166	0.83723085	F
541	1.58529785	F	382	0.843245083	F
13	1.58228085	F	647	0.896118767	F
289	1.576070417	F	633	0.914801133	F
855	1.561209717	F	732	0.9306929	F
37	1.531535967	F	56	0.938088217	F
475	1.513334433	F	824	0.939480767	F
781	1.51170545	F	85	0.94932655	F
208	1.5045619	F	706	0.9574779	F
424	1.49505355	F	979	0.699354883	F
381	1.494195283	F	981	0.87720725	F
543	1.48877365	F	982	0.951431767	F
958	1.484316217	F	987	0.83956475	M
38	1.4731066	F	989	0.746570083	M
113	1.816156317	M	719	0.616539367	M
170	1.809003817	M	705	0.759629167	M
361	1.804365067	M	321	0.76830645	M
800	1.782337083	M	954	0.77304995	M
246	1.7312373	M	595	0.773267083	M
230	1.7032764	M	877	0.774099683	M
335	1.701781467	M	593	0.774585733	M
564	1.692312567	M	129	0.776378817	M
7	1.6485943	M	569	0.832791767	M
3	1.6379044	M	711	0.8521944	M
224	1.626050667	M	622	0.859333383	M
194	1.598008517	M	521	0.8922438	M
112	1.5888819	M	736	0.894005	M
175	1.586977067	M	159	0.895707067	M
313	1.57841465	M	133	0.912036867	M
1061	1.5783577	M	207	0.92464725	M
126	1.559469517	M	423	0.9307243	M
397	1.5522724	M	512	0.934638567	M
