# Supplementary material for: Unveiling the Molecular Mechanisms Regulating Muscle Elasticity in the Large Yellow Croaker: Insights from Transcriptomics and Metabolomics
Source: Int J Mol Sci. 2024 Oct 11;25(20):10924. doi: 10.3390/ijms252010924 (PMC11507341; doi:10.3390/ijms252010924)
Supplement: Supplementary file 1 [file ijms-25-10924-s001.zip › Table S2.pdf]

**Table S2.** Sequences of Primers used for PCR data validation of large yellow croaker (*L. crocea*) transcriptomic.

| Gene Name | Forward Primer (5′-3′) | Reverse Primer (5′-3′) |
|---|---|---|
| *β-actin* | CTACGAGGGTTATGCCCTGCC | TGAAGGAGTAACCGCGCTCTGT |
| *spdya* | TTTCACACCCGCTTACCTCC | CATCCCCTGATGGGAACCTC |
| *hipk3* | TTTTTCCGAAGGAAGGGCCG | TTTCTGCCTTGGTGTCCTCG |
| *gabrr1* | CAAGCACCAACAACCAGAGC | TAAAGGAGCGCTTGGAGTGG |
| *myo5c* | ACAAGTCCACTGACGACGAC | AACTTTGCGGACAAACGACG |
| *scn1bb* | CAACGACACGGGAACCTACA | CCACCACAGAGAGTTGGACC |
| *tent5bb* | TTCTTGCCTGAAGGGGTGTG | TCCACATTCTTGCCGGTGTT |
| *gpnmb* | ACGCCACAAAGGTAAACCCA | CAGCTCCGTACCATCCTCAC |
| *tmem144b* | ACTCCAGAGCCATCCTACCA | GGTCAGCAAGGAACCAGTCA |
| *sh2d3ca* | CTGGATGTGTGCCCGAAGAT | ACATAGCTGGCAGTTCGAGG |
| *wfikkn2a* | GTGGTGGCAACGAGAACAAC | GAAGAGTCACCTCCAAGGGC |
| *csrp3* | GCAGGAAAGCCCTGGCATAA | GTCCTTGTCTGTCACTGTGGT |
| *mok* | ATTGGCGTGAAAAACAGGGC | TTAGGCGGCGGATTGTTCTT |
| *acsl5* | TCAACCGCGTCTACGACAAA | CACCTCGGCGTATTTCCTGT |
| *mat1a* | CCAGAAGAGGGTGCTCAAGG | GACAAAGCGACCACTAGGCT |
| *pitpnc1a* | ATCTTTGGCAGTGAGCCCAG | GTTCCACACGTGTTTGCAGG |
| *Sybu* | AGTCTGTGTCCCCCTCCAAA | CTGAGCTGAAAGAGAGGCCC |
| *Crhbp* | GTTGCGGAGAATCGGGAGAT | TTGTCGCAGCCGATCTTCAT |
| *Polh* | TGACTTGTGTCGGGGGATTG | CTCTTCAAGCTCAAGGGCCA |
| *dydc2* | ACTCAACTGCCGACCAAGAC | GTCAGCGGAACGAGGACTTT |
| *adamts13* | TCCATTGGTAGAGAAAGACACAGT | CGCCCAAGGTCATGTCTCTC |
| *slc6a14* | AGCTGATGAGAGCAGAGGGA | ATCCAGGTAGGGATGCCACT |
| *pklr* | TGGACATAGACCAGGAGCCA | TGGTATTCGTGAGAGCCGTG |
| *neto2b* | AGTGGGAGTCGTGAGGATGT | CAGGGTGGGTCTGCAAAAGA |
| *hspb9l* | CCAGCCCCGATAACCATGAG | CCCGTCGAGGATGAAGTCTG |
| *pcdh20* | GGTTACGGTGAGATCGGCAT | AGATGTCACTGCCGTTGAGG |
| *hipk3* | ACTGATCGACTTCGGCTTGG | CGGCCCTTCCTTCGGAAAAA |
| *inhbb* | TTTTGTGTTTAGTTGCGTGCCT | GGGATGGGTGATGTTGGGTC |
| *dpr* | CTGAAAACAGCCTCCTGGGT | TGGGGTTGGGAATAGGGACA |
| *sds* | CACCTGTGCAAAACTTGGGC | TTCAGCCTCTCCACTGTTGG |
| *sytl1* | CTGTTCAGCTCGGGGGATTT | ATGGGTCAGAGCGGTTCTTG |
| *dydc2* | ACTCAACTGCCGACCAAGAC | GTCAGCGGAACGAGGACTTT |

| | | |
|---|---|---|
| *adamts13* | TCCATTGGTAGAGAAAGACACAGT | GCCCAAGGTCATGTCTCTCA |
| *umodl1* | GTACACTGCCAAGCCAGGAT | GCGGACATCATCTGCTCGTA |
| *myo5c* | ACAAGTCCACTGACGACGAC | AACTTTGCGGACAAACGACG |
| *tspan13b* | CCTGTGACGCTGCATGTTTT | ATCCCTCCTACGAAGCGGAG |
| *myl13* | TACTTACGGCCAGTGTGGTG | TTCAGCCTTGGGCTTTCCAA |
| *ndnf* | ATGATGCCTTCATGCTCGCT | TGTACGCCCGTGAGATCTTG |
| *pthlha* | GACGACACGAGCTCTGATACA | GAACAGAGCGAAGCACCACT |
| *myl2b* | GCCCAGATCCAGGAGTTCAAA | AAACGGCCTAAAGCTGCGAA |
| *fabp2* | TTCAACGGAACCTGGAAGGTC | CCCGTCTGTTCGATGGTGAC |
